# Tirzepatide: a dual-action solution for obstructive sleep apnea and obesity

**DOI:** 10.1097/MS9.0000000000002975

**Published:** 2024-12-12

**Authors:** Zain Afridi, Kanza Farhan, Fnu Fahad, Malik W.Z. Khan, Izere Salomon

**Affiliations:** a Khyber Medical College, Peshawar, Pakistan; b Jinnah Sindh Medical University, Karachi, Pakistan; c Yale University School of Medicine, New Haven, CT, USA; d University of Rwanda College of Medicine and Health Sciences, Kigali, Rwanda

*Dear Editor,*

Obstructive sleep apnea (OSA) and obesity are two common and frequently related health disorders that substantially influence people’s lives. Given their frequent co-occurrence, the search for effective therapies targeting both disorders is crucial. This editorial highlighted Tirzepatide, a novel dual agonist for glucose-dependent insulinotropic polypeptide (GIP) and glucagon-like peptide-1 (GLP-1), promising in treating both OSA and obesity, potentially offering a dual-action therapy^[1,2]^.

## Background

OSA is defined by recurring bouts of upper airway blockage during sleep, which results in decreased airflow and increased carboxyhemoglobin levels for 10 seconds or longer. This illness is more prevalent in men and older persons, with an estimated 24 million untreated instances in the United States. Symptoms include excessive daytime sleepiness, loud snoring, and morning headaches. OSA relates to several health hazards, including cardiovascular and respiratory difficulties, and is measured using the Apnea-Hypopnea Index (AHI). Current therapies include weight loss, surgical procedures, positive airway pressure (PAP), and mandibular advancement devices (MADs), each with limitations such as poor adherence and uncertain cardiovascular outputs^[1,2]^.

## Mechanism of action and pharmacokinetics

Tirzepatide is a synthetic peptide based on GIP, which acts as a receptor agonist (RA) for both GIP and glucose-like peptide-1 (GLP-1)^[3-5]^. Being GIP and GLP-1 RA, tripeptide is approved to manage type 2 Diabetes Mellitus (T2DM)^[5]^. It is administered as a subcutaneous (S/C) injection, typically once a week, to ensure steady therapeutic levels. It acts on both GIP and GLP-1 pathways as a dual receptor agonist. Clinical trials revealed that Tirzepatide can be used not only for managing T2DM but also for reducing weight^[3-7]^ and biomarkers, which are associated with cardiovascular complications such as inflammation and endothelial dysfunction^[4,5,7]^. The GIP pathway plays a role in fat metabolism and insulin sensitivity, while GLP-1 reduces appetite and promotes satiety. Together, these mechanisms lead to substantial weight loss, reduced adiposity, and improved metabolic outcomes. Leptin, a fat molecule secreted by adipose tissue, which is associated with obesity, is also reduced by Tirzepatide^[7]^. In some cases, Tirzepatide is also used to significantly lower systolic and diastolic blood pressure (BP) in the Japanese population, at about 11 and 5.6 mmHg, respectively^[3]^. Although tripeptide is significantly effective in managing HbA1c and BP, there are few adverse effects reported in its use, which include common gastrointestinal side effects such as nausea, diarrhea, vomiting, and some rare cases of injection site reaction and pancreatitis^[4-6]^.

## Comparison with other therapies

While other GLP-1 receptor agonists like liraglutide also promote weight loss, Tirzepatide demonstrates superior efficacy in head-to-head trials, achieving up to 20% body weight reduction. Liraglutide, an FDA-approved GLP-1 analog with 97% similarity to human GLP-1, has been recently approved for marketing to treat obesity at a 3.0 mg dose. It reduces appetite and energy intake. This mechanism makes a significant impact on reducing weight and AHI. These results show that lowering weight improves the AHI score^[8]^. Furthermore, the combination of phentermine and topiramate, approved for weight loss, shows some improvements in OSA by reducing AHI scores. However, these medications come with limitations, such as safety concerns and gastrointestinal side effects^[9]^

## Clinical evidence supporting dual-action benefits

As recent research shows, patients with OSA can benefit from Tirzepatide’s effectiveness in helping them lose weight. Eli Lilly and Company conducted a clinical trial with 469 participants with OSA and a BMI of more than 30 kg/m^2^. Tirzepatide significantly reduced body weight and improved AHI in the trial compared to a placebo. In Study 1, which excluded participants using PAP, Tirzepatide demonstrated a significant improvement compared to placebo at the end of 52 weeks. It reduced AHI by over half compared to the placebo and body weight by one-fifth of the baseline. In the second study of the same trial, which included participants who were also using PAP along with Tirzepatide, the Tirzepatide group showed ten times more reduction in AHI and body weight than the placebo group. Based on these findings, Tirzepatide may be able to treat OSA and obesity successfully^[8]^.

The introduction of Tirzepatide may alter treatment paradigms for OSA and obesity. Patients may benefit from enhanced quality of life and fewer comorbidities, and healthcare providers may consider Tirzepatide a potential treatment choice. However, cost-effectiveness and accessibility are still challenges. Ongoing research is required to investigate combination medicines and their long-term safety and efficacy. Furthermore, Tirzepatide potential usefulness in additional metabolic illnesses requires further investigation.

## Limitations and future directions

Despite these promising results, several challenges remain. Long-term safety data on Tirzepatide is still limited, and gastrointestinal side effects such as nausea, vomiting, and diarrhea have been reported. Rare cases of pancreatitis and injection site reactions have also been documented. Additionally, the high cost of Tirzepatide may limit its accessibility, posing a challenge for widespread clinical adoption. Further research is needed to explore Tirzepatide’s effects on cardiovascular outcomes in OSA patients and its potential role in other metabolic disorders. Investigations into combination therapies with PAP or other weight-loss medications could provide deeper insights into optimizing treatment strategies.

## Conclusion

Tirzepatide represents a possible dual-action treatment for OSA and obesity. Further study and innovation are required to properly grasp its benefits and incorporate it into therapeutic methods. Healthcare practitioners and academics should prioritize investigating Tirzepatide’s potential to improve patient outcomes while addressing the enormous problems posed by OSA and obesity

## Data Availability

No data was generated for this manuscript.
